# Key changes to improve social presence of a virtual health assistant promoting colorectal cancer screening informed by a technology acceptance model

**DOI:** 10.1186/s12911-021-01549-z

**Published:** 2021-06-22

**Authors:** Melissa J. Vilaro, Danyell S. Wilson-Howard, Mohan S. Zalake, Fatemeh Tavassoli, Benjamin C. Lok, François P. Modave, Thomas J. George, Folakemi Odedina, Peter J. Carek, Janice L. Krieger

**Affiliations:** 1 STEM Translational Communication Center (STCC), University of Florida, Weimer Hall 2043, PO Box 118400, Gainesville, FL 32611 USA; 2 Bethune-Cookman University, Daytona Beach, USA; 3 Computer and Information Science and Engineering, University of Florida, Gainesville, USA; 4 Health Outcomes and Biomedical Informatics, University of Florida, Gainesville, USA; 5 Hematology and Oncology, University of Florida, Gainesville, USA; 6 College of Pharmacy and College of Medicine, University of Florida, Gainesville, USA; 7 Community Health and Family Medicine, University of Florida, Gainesville, USA

**Keywords:** Technology acceptance model, Colorectal cancer screening, Web-based intervention, Virtual agent, Rural health

## Abstract

**Background:**

Understanding how older, minoritized patients attend to cues when interacting with web-based health messages may provide opportunities to improve engagement with novel health technologies. We assess acceptance-promoting and acceptance-inhibiting cues of a web-based, intervention promoting colorectal cancer (CRC) screening with a home stool test among Black women.

**Materials and methods:**

Focus group and individual interview data informed iterative changes to a race- and gender-concordant virtual health assistant (VHA). A user-centered design approach was used across 3 iterations to identify changes needed to activate cues described as important; such as portraying authority and expertise. Questionnaire data were analyzed using non-parametric tests for perceptions of cues. Analysis was guided by the Technology Acceptance Model.

**Results:**

Perceptions of interactivity, social presence, expertise, and trust were important cues in a VHA-delivered intervention promoting CRC screening. Features of the web-based platform related to ease of navigation and use were also discussed. Participant comments varied across the 3 iterations and indicated acceptance of or a desire to improve source cues for subsequent iterations. We highlight the specific key changes made at each of three iterative versions of the interactive intervention in conjunction with user perception of changes.

**Discussion:**

Virtual agents can be adapted to better meet patient expectations such as being a trustworthy and expert source. Across three evolving versions of a Black, VHA, cues for social presence were particularly important. Social presence cues helped patients engage with CRC screening messages delivered in this novel digital context.

**Conclusions:**

When using a VHA to disseminate health information, cues associated with acceptability can be leveraged and adapted as needed for diverse audiences. Patient characteristics (age, identity, health status) are important to note as they may affect perceptions of a novel health technologies *ease of use* and *relevancy* according to the leading models.

## Introduction

### Technology, cancer screening, and an aging population

With the ongoing expansion of telemedicine for use among rural and aging populations [1, 2], contextualizing facilitators and barriers of health technology use and non-use is needed. Despite this need, systematic exploration of features predicting acceptance and use of health technologies among aging, rural, and minoritized populations is lacking [3–5]. The Technology Acceptance Model (TAM) is a framework for establishing acceptability and usability of technology [6]. TAM relies on two constructs; perceived usefulness (e.g., can the technology enhance my performance) and perceived ease of use (e.g., will using the technology be low effort). An adapted version, the Senior Technology Acceptance Model (STAM) incorporates the role of individual characteristics which can include health conditions, cognitive ability, or physical functioning to determine how older adults use technology for health [7]. These frameworks are considered the most relevant to exploring patient perceptions of health technologies among computer scientists developing health applications [8].

Technology may play an important role in enhancing access to colorectal cancer (CRC) screening for at risk adults. CRC is the 3rd leading cause of cancer deaths among US adults [9]. Although all adults at average risk of CRC should begin regular screening at age 50, screening disparities exist. Historically, Black adults have lower CRC screening rates compared to White adults. Despite declines in Black-White disparities in late-stage diagnosis, incidence in CRC remains higher among Black adults [10]. In addition to race, there are significant geographic inequalities. Rural adults are also less likely to obtain screening within guidelines compared to urban counterparts [11, 12]. The Fecal Immunochemical Test (FIT) is a non-invasive, low-cost, acceptable, accurate screening modality for adults at average risk and may help improve screening rates if offered over colonoscopy [13]. As the FIT can be completed at home and mailed to a lab for processing, it has potential to enhance access to and ease of screening.

### Virtual human agents as a source of acceptable cancer communication

Innovations that supplement and support patient care may be an important tool for improving communication and access to needed cancer screenings. One emerging tool is the use of virtual agents, which are customizable characters that allow for interactive dissemination of health communication. Virtual agents may facilitate informed decision-making, enhance trustworthiness, and improve patient engagement with health [14, 15].

It is well documented that a healthcare provider recommendation is a strong predictor of compliance with CRC screening [16–18]. Thus, if deemed a credible source, virtual health assistants (VHAs) may become a scalable, effective strategy to communicate cancer screening messages. Furthermore, VHAs may simultaneously support other public health goals. One objective of the Healthy People 2030’s Health Communication and Health Information Technology Workgroup is to decrease the proportion of adults who report poor communication with their health care provider [19].

### Social presence

Social presence, the general sense of being with another person, is important when using VHAs to communicate with patients [20]. Through the lens of TAM, social presence is relevant to patients accepting VHAs in part because perceptions o f social presence can lead to a desire for future interaction [21]. Patients may perceive a virtual agent in a number of ways including warm [22], interested, friendly, or emotional about a conversation [23]. In previous work, cues associated with social presence promote a sense of acceptability and improved message engagement [24, 25].

The purpose of this paper is to describe specific design features (e.g., cues) adapted to improve acceptability and useability of a web-based intervention promoting CRC screening. The intervention is delivered by a race-and gender-concordant VHA and covers all screening modalities with a focus on FIT. This paper builds on previous work related to this project describing a user-centered design (UCD) process and analysis of cues [26–28]. Exploratory triangulation of qualitative and quantitative data contextualizes the impact of changes. We answer the following questions:

1. What acceptance-promoting and acceptance-inhibiting cues are identified in a VHA-delivered intervention promoting CRC screening?
2. What key changes improved patient perceptions of the intervention?

## Methods

### Study overview

A convenience sample of non-Hispanic, Black women from a largely rural region of the southern U.S. participated in focus groups and individual think-aloud interviews between January 2017 and November 2018. Participants were recruited via flyers and community engagement as part of a larger study. Eligible participants were between 50 and 73 years old and proficient in English. Trained moderators facilitated a semi-structured discussion that was audio-recorded and transcribed. Participants also reported perceptions of the intervention, attitudes, behaviors, and demographic characteristics on paper questionnaires. Trained research assistants later entered questionnaire data into Qualtrics™ [29].

Participants viewed print prototypes (e.g., focus groups 1 and 2) or tested one of three iterations of the intervention on mobile Samsung Galaxy phones with headphones. Participants then discussed their perceptions of the prototypes and last filled out a paper questionnaire. Based on approved procedures from institutional review boards, prior to participation all individuals provided written informed consent. Participants received a $35 gift card. All procedures were approved by Institutional Review Board, IRB201601642. All methods were carried out in accordance with relevant guidelines and regulations.

### Data analysis

For qualitative data, we conducted thematic analysis of patient comments using comparative analysis across all versions tested (print prototypes, interactive version 1, interactive version 2, and interactive version 3) (Table 1). Interactive versions included verbal information provided by the virtual health assistant, closed-ended questions, and nonverbal behaviors with animated motion. Trained raters coded transcripts and attained acceptable inter-rater reliability, described elsewhere in a paper that describes participant reactions to screening and the VHA [27]. The focus of this paper is to detail how participant comments were used to inform and alter specific development aspects of the VHA across successive iterations. Team members discussed and tracked when changes were made to the VHA and intervention. This process allowed for a detailed cataloging of the specific key adaptations made throughout the UCD process [30].

**Table 1:** Participant characteristics and group type

Group type	Stimuli	N	Age (mean, SD)
Focus groups			
1	Print prototypes	5	64.2 (3.4)
2	Print prototypes	8	60.8 (6.9)
3	Interactive version 1	7	55.8 (5.0)
4	Interactive version 1	4	55.7 (7.2)
5	Interactive version 2	7	60.0 (2.0)
6	Interactive version 2	1	60
7	Interactive version 3	11	62.1 (6.3)
8	Interactive version 3	4	59.7 (5.5)
Think-aloud interviews			
1–6	Interactive version 3	6	63.5 (3.4)
Total		53	60.9 (5.5)

Focus groups #4 and #6 each had one non-black participant, not represented in total N. Groups #2, #3, and #5 are each missing one response for age from the questionnaire data

While qualitative data was the primary driver of the iterative changes throughout the design and evaluation process, we conducted post-design analysis of the quantitative data collected via paper questionnaires. This post-design analysis served two goals; one to assess if questionnaire data was consistent with the insights derived from qualitative data and two to provide additional insights regarding when discernable improvements may have been perceived, across the three interactive versions. While all participant comments informed the intervention, only participants who tested interactive prototypes (i.e., version 1, 2, and 3) are included in exploratory statistical analysis of perceptions. Participant responses to questionnaire items were grouped in accordance with the version tested to facilitate comparisons across the three interactive versions. Overall, descriptive statistics were computed, independent-samples Kruskal–Wallis non-parametric tests were conducted on participant characteristics.

Perceptions of the VHA and the web-based app were analyzed to assess distributions of scores on questionnaire items adapted from validated measures. Responses reported on a Likert Scale (1 = strongly agree to 5 = strongly disagree) were analyzed with the independent-samples nonparametric median tests as exploratory triangulation of qualitative data. One item was selected for each qualitatively derived cue. Statistical significance was set at ≤ 0.05 after adjusting for multiple comparisons. When a significant main effect was found, post-hoc pairwise comparisons were conducted, also adjusted for multiple tests. Analysis was conducted using IBM SPSS Statistics™ [31].

## Results

Fifty-three non-Hispanic, Black women participated (Table 2). Marital status was similar across groups, however, participants who tested version 1 reported higher levels of education and income than those who tested version 3, *p* < 0.05.

**Table 2:** Participant characteristics

Age (mean, standard deviation)	60.9 (5.5)
Race	**(N, %)**
Black/African American	53 (100%)
Gender	
Female	53 (100%)
Marital status	
Married	13 (24.5%)
Divorced/separated	15 (28.3%)
Single	19 (35.8%)
Widowed	6 (11.3%)
Employment	
Full-time or part-time for pay	18 (34%)
Retired	17 (32%)
Unable to work due to disability	9 (17%)
Unemployed	6 (11.3%)
Volunteer or prefer not to answer	3 (5.6%)
Income (2016)	
< $20,000	19 (35.8%)
$20,000–$74,999	13 (24.5%)
≥ $75,000	2 (4%)
Prefer not to answer	19 (35.8%)
Education	
≤ High school/GED/trade school	28 (52.7%)
Some college or college grad	19 (35.8%)
Postgraduate training	3 (5.6%)
Prefer not to answer	3 (5.6%)
Total participants	53 (100%)

### Qualitative results: perceptions of source cues and navigability

Table 3 details specific key changes, and when they were made throughout the development process. Key changes addressed various cues within the domains of social presence, trust, expertise, and navigability, which overall are likely to influence acceptance and ease of use which are key components of TAM.

**Table 3:** Key changes to acceptance-inhibiting cues to promote perceptions of VHA acceptability

Cue	Key themes	Key changes	Version
Clothing	Not professional (e.g., scrubs)	Updated the VHA so she was dressed in business casual clothing rather than scrubs	Print to V1
Real/Fake	Low quality, animated, robotic looking	Changed lighting to introduce depth in visual features	V.1 to V.2
Scary/Creepy	Looked like a vampire (e.g., "fangs")	Added shading to mouth and teeth	V.1 to V.2
Expertise	Preference for doctor vs. nurse or lay health worker	Added white coat (e.g., business casual was too casual)	V.1 to V.2
Authority	Preference for middle age (e.g., too young = not enough knowledge vs. too old = not enough current knowledge)	Removed gray hair	V.1 to V.2
Trustworthiness	Desire for accurate, relevant information, desire to not feel targeted	Removed color-coded response options (e.g. red = “no”) to prevent perception of judgment when answering questions	V.1 to V.2
Navigability	Perceptions of how easy it is to use and navigate through the app	Added pause button with ability to tap to pause. Updated text size of subtitles. Removed user transition from waiting room to clinic room	V.1 to V.2
Movement	Unnatural movements, excessive hand gestures and rocking	Used motion capture suits to update motion to correspond with script (e.g. breathing animation)	V.2 to V.3
Appearance	More feminine, more dignified	Changed hairstyle, added jewelry	V.2 to V.3
Friendliness/likability	Angry looking, stressed out, not approachable	Added smile, removed furrowed brow	V.2 to V.3
Interactivity	Poor eye contact, low interactivity, limited opportunity to ask questions or have responses tailored to personal needs	Focused eye gaze, added randomness in eye movements (e.g., static to dynamic), new response option for health behavior questions (e.g., “yes, occasionally”), reduced extra info in VHA script	V.2 to V.3
Voice	Reading from a script, too fast/ loud, persuasive intent, subtitles not synched with audio	Selected race and gender concordant voice, adjusted subtitle speed, hired professional voice actors to record script, presented options to users	All

#### Key changes to improve social presence

**Fig. 1:**
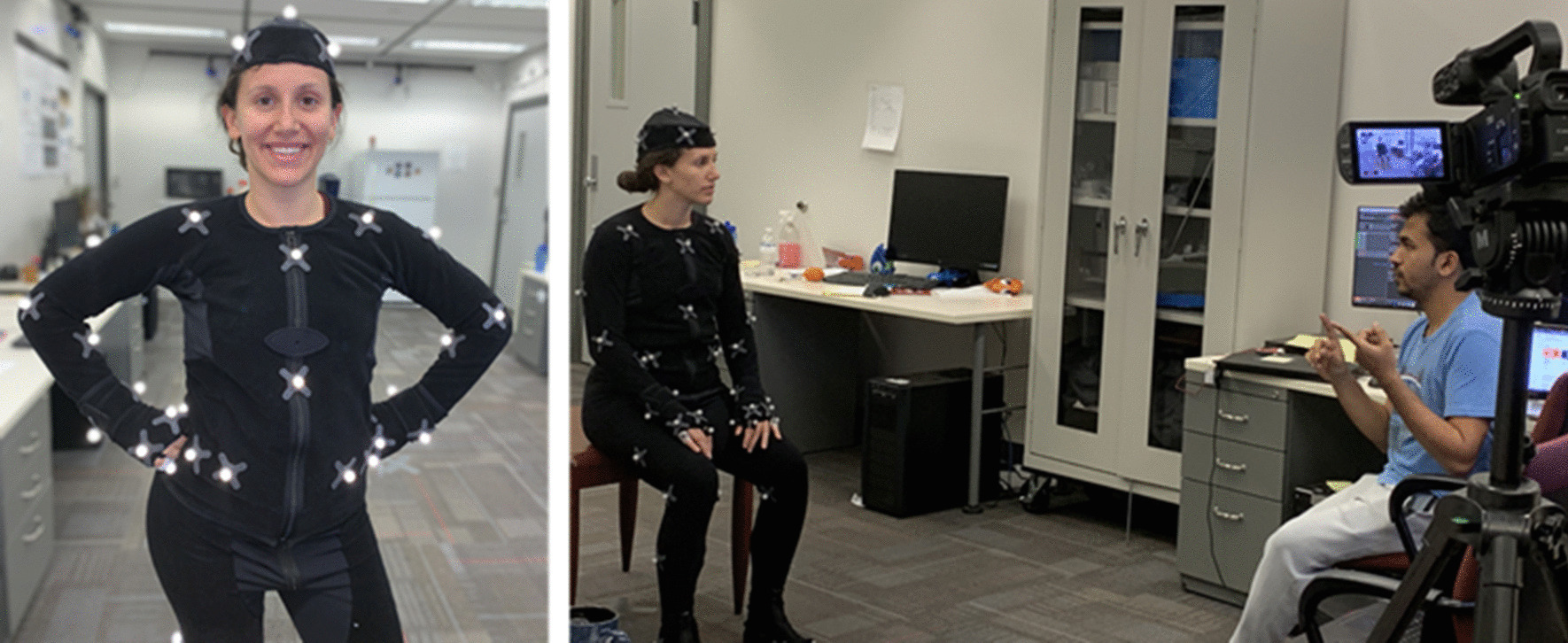
An actor wears a motion-capture suit as they prepare to read the scripted text of the intervention while their gestures are recorded in a session with research team member and co-author (MZ)

Social presence improved with modifications to cues related to perceptions of the VHAs (1) movement, (2) being real versus fake, (3) being scary, and (4) ability to provide an interactive experience. Comments indicated that changes improved perceptions of these cues across versions. For example, when social presence was low participants said things like “…computers don’t always understand, or they’ll only have the information that was inputted to them, therefore, it’s not like a thinking person. I’d almost prefer a person that could think and analyze things, and dissect it.” (P103, FG2, print). Looking away from the VHA was an indicator of low social presence, “…I got where I quit looking at her doing it,” (P113, FG5, VER2) and “…I just looked at that little thing… [laughter], after that I didn’t look.” (P46, FG4, VER1). When social presence was high participants said things like, “It’s like she was talkin’ to a live person when she’s talkin’ to us…” (P136, FG7, VER3). Motion capture was one technique used to improve perceptions of movement (Fig. 1).

Another cue strongly related to social presence were perceptions of the VHA being real or fake. By version 2, comments started to reflect a mix of participants thinking the VHA was real “for the most part she looked real” (P118, FG5, VER2) and questioning her realness “Why wasn’t it a real person, real doctor?” (P114, FG5, VER2). By version 3, the VHA is described almost exclusively as real, *“*she did a beautiful job. One time, I couldn’t even think that she wasn’t a real person because of her wisdom that was comin’ from her and from the test, and it did a very good job on that.” (P132, FG8, VER3).

#### App development to enhance trustworthiness

Trust included cues related to the VHA’s voice, friendliness, and appearance. While some participants testing early versions were reluctant to interact with the VHA, others indicated they would trust the VHA even though they don’t typically trust doctors. This trust was due to the perception that the VHA, as a computer, had additional access to information which could be relayed without bias. With adaptations, aspects of trust were communicated when participants indicated they appreciated not feeling like a targeted population by the VHA. Comments included; “She didn’t discriminate. Some people wound up doin’ those things. They say, “Well, are you through the age between da, da, da? Are you Black? Are you Hispanic?” That was never…” (P137, FG7, VER3). Allowing women to feel communicated with versus targeted because of personal characteristics that are also non-modifiable risk factors, was an important cue; “I liked it because she didn’t put no color in there, no age, none of that. What she was sayin’, she was sayin’ everybody.” (P138, FG7, VER3). Another participant commented, “At one time, I thought I was bein’ singled out, but no, this is goin’ on with people all over the world” (P135, FG8, VER3).

#### Expertise

**Fig. 2:**
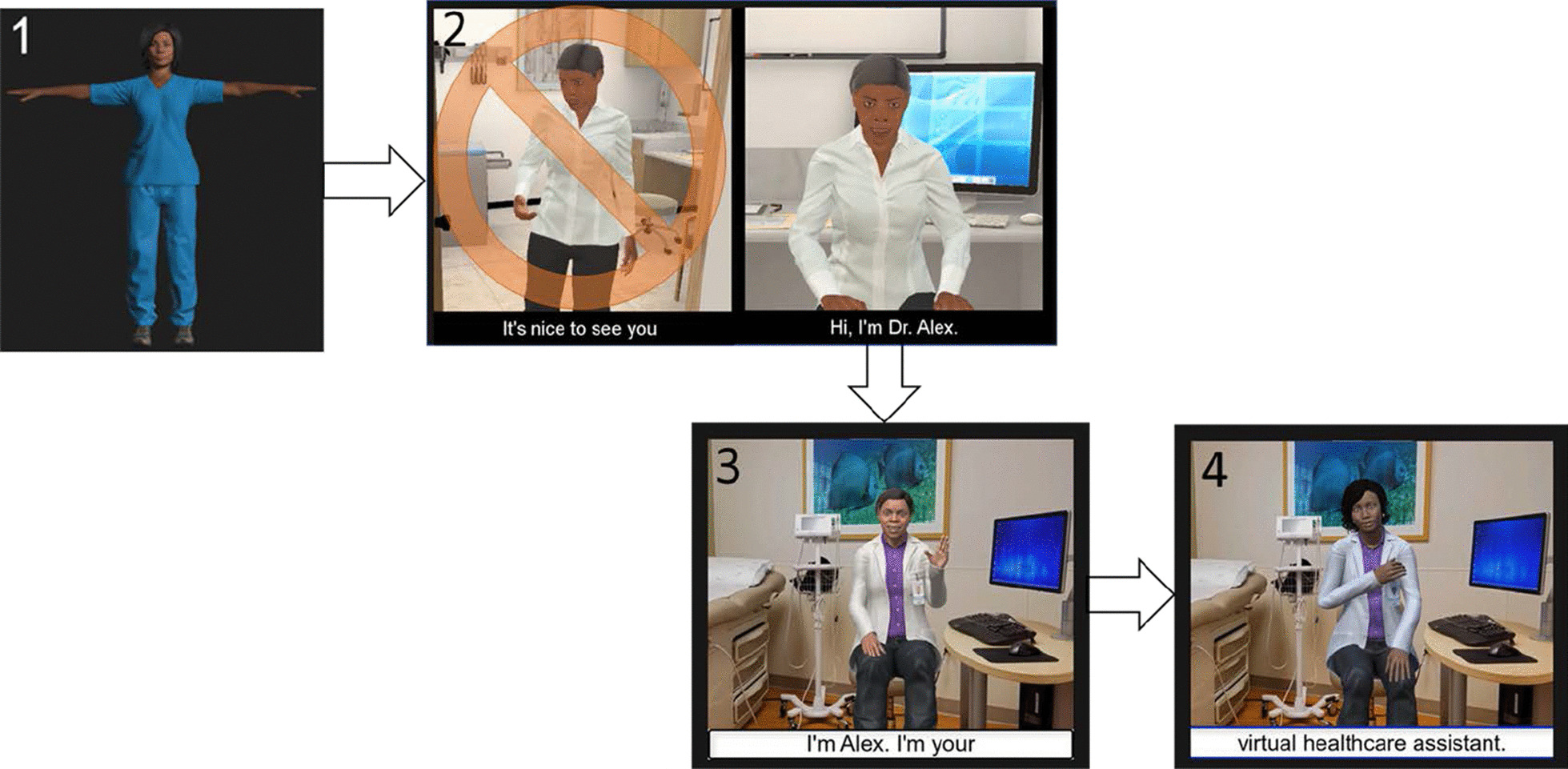
Overview of design changes. (1) Selected example of print stimulus presented to participants on printed sheets of paper in early data collection sessions. (2) Screen shots from the animated and interactive versions of the virtual health assistant (VHA) delivering colorectal cancer screening messages. These interactive versions were tested on mobile phones. Images show design changes across the three interactive versions. Interactive version 1 eliminated an introduction showing a closed door and the VHA opening to greet user, then walking back to her chair (image 2). Interactive versions 2 and 3 (images 3 and 4) show updates to the clinic room environment, attire, and appearance among other updates detailed in Table 3

The VHA being perceived as a medical authority, attire, and age of the VHA influenced perceptions of expertise. Women liked that she looked professional in medical attire, “That’s a professional without a suit.” (P101, FG2, VER1). Changes such as adding a name badge, updating clothing to include a white medical coat, and adjusting perceptions of the VHA’s age improved evaluations of expertise across versions (Fig. 2).

#### Navigability

The ease at which users could move through the intervention was important as indicated by one participant whose app unexpectedly paused during use commented, “I was getting a little antsy because this kept dropping, but I stuck with it because I know how important this is.” (P113, FG5, VER2). All versions of the app contained a pause button. Versions 2 and 3 were adapted allowing participants to pause the interaction by tapping the mobile phone screen and resumed by clicking a play button. Although indicated as a preference, no version contained a rewind or fast-forward option, as these were hypothesized to interfere with validity during future testing in the clinical trial phase. Audio narration was subtitled throughout, however versions 2 and 3 featured larger subtitle text size. A chat log was eliminated after version 1, as participants did not find it useful. Additionally, a waiting room scene was removed based on feedback, “if you go straight to the [VHA], I think you would get more people to use it. Don’t take them through the step of waiting in a waiting room…because honestly, if I go there and they’re like, you’re number such and such, you need to sign in… I’m not doing it.” (P35, FG3, VER1).

### Quantitative results: perceptions of VHA and application

Analysis of questionnaire items representing relevant domains of change, revealed two statistically significant items (Table 4). An independent-samples median test indicated responses to “*The virtual humans gestures distracted me as I tried to listen*” were significantly different across the iterations tested, *H*(2) 12.102, *p* = 0.002. Post-hoc analysis with a Bonferroni correction for multiple tests indicate, version 2 was significantly more distracting than version 3 (*p* = 0.002). Version 1 was not significantly different from Version 2 (*p* = 0.144) or Version 3 (*p* = 0.648). This difference was only found for the reverse scored question. Additionally, responses to “*The virtual human looked like an expert*” were significantly different across iterations, *H* (2) 6.078, *p* = 0.048. Post-hoc analysis revealed that version 3 was perceived as more expert than version 2 (*p* = 0.023). However, when adjusted for multiple tests with the Bonferroni correction, differences between version 2 and 3 were no longer significant (*p* = 0.068). No other items were significantly different.

**Table 4:** Results of non-parametric median test comparing user perceptions by version tested

Parameter	Median (Q1, Q2)			Statistics		N
	Version 1	Version 2	Version 3	H (a)	P value	
The virtual human looked like a doctor	2 (2,4)	2.5 (1.3,4)	2 (2,2.5)	2.057	0.358	39
There was too much inconsistency in this application	4 (4,4)	4 (1.75, 4)	4 (4,5)	4.150	0.126	39
The application was easy to use	2 (1,2)	1.5 (1,2)	2 (1,2)	1.660	0.435	39
The virtual human looked very realistic	2 (2,4)	3 (2,4)	2 (2,3)	0.503	0.778	33
The virtual human had a pleasing voice	2 (2,2)	2 (2,2)	2 (1,2)	3.097	0.213	32
The virtual human's gestures distracted me as I tried to listen (reverse scored)	2 (1,2)	3.5 (2,4)	2 (1.5,2)	12.102	0.002*	40
The virtual human's gestures distracted me as I tried to listen	4 (4,5)	2.5 (2,4)	4 (4,4.5)	0.623	0.732	40
The virtual human looked trustworthy	2 (2,3)	2 (2,3)	2 (2,2)	2.665	0.264	41
The virtual human looked like an expert	3 (2,3)	3 (2,3)	2 (1.5,2)	6.078	0.048*	39

^*^Statistical difference detected between groups (*p* < .05) with sig. values adjusted by Bonferroni for multiple tests; (a) the test statistic adjusted for ties. 1 = strongly agree and 5 = strongly disagree

## Discussion

VHAs were adapted to mimic desired social and physical cues associated with acceptability. The UCD process allows for team and user feedback, in a timely workflow. This allows for quick and systematic development of the technology. A number of changes that influenced acceptance and useability of a VHA-delivered screening intervention among Black women are discussed. Cues related to social presence, trustworthiness, expertise, and navigability were important. Participant description of these cues informed changes to sequential iterations of the intervention. While this paper focuses on the specific topic of colorectal cancer screening, the process of systematically and iteratively engaging community members in design decisions for web-based interventions can be applied to many health-promotion goals and topics. Using adaptive technology-based intervention design strategies has promoted sustained participant engagement various populations [32].

Exploratory analysis of questionnaire data revealed significant differences in perceptions of some cues across the 3 versions tested, which correspond with the timeline of key changes based on participant feedback. For example, changes made to improve perceptions of VHA movements (e.g., I was distracted by the VHAs gestures) between version 2 and 3, were confirmed successful with statistically significant differences (improvement) found between version 2 and 3 on Questionnaire data.

Consistent with the literature [33–36], our results confirm STAM and the UCD process are effective ways to ensure patient expectations are met (e.g., attaining the right level of relevant cues) in an efficient timeframe. This is an important consideration as the role of technology in health promotion continues to expand. In fact, new definitions of health literacy include expectations that organizations assume responsibility for ensuring materials are easy to use, in contrast to previous definitions of it as an individual characteristic [37]. Reframing health literacy from individual deficit to organizational responsibility is well aligned with a UCD approach. For one, a UCD takes responsibility for engaging individuals in the process of creating effective and usable health materials. Thus, a deficit model of individual failure is less likely to be applied to individuals who do not meaningfully engage with content.

### Social presence

Previous work demonstrates when unfamiliar with a platform (e.g. mobile application or website) people rely more on traditional cues and less on information requiring experience with the platform [38]. Humanlike qualities can help virtual agents seem more familiar. Comments suggesting the VHA was fake and/or scary indicated that early versions of the VHA may have had insufficient social presence. However, when users perceived the VHA as a computer with additional access to information, a perceived benefit of the VHA was it could provide reliable, unbiased information more efficiently than a human. The VHA being “not fully human” may create a sense of trust and freedom to circumnavigate inherent biases and racism that can shape communication of health information for Black and minoritized populations. Additionally, apprehension about collecting stool and the invasive colonoscopy modality are documented factors that lead to poor screening. The VHA may have also contributed to enhanced engagement as a trusted source of information who could provide visual demonstrations of stool collection to actively address barriers.

### Trustworthiness

Trust is an essential component of health communication that has special considerations when technology is used. Questionnaire data indicated that overall, all versions were considered trustworthy. Qualitative data provide additional insights on trust. Specifically, patients felt positive about not being targeted by the VHA. Previous research confirms feeling categorized based on risk factors can produce a sense of hopelessness, especially when risk factors are non-modifiable (e.g., age, race, genetic factors) [39, 40]. These findings suggest the VHA avoided making women feel labeled as a risky population. That a culturally-tailored VHA does not make Black women feel targeted has important implications for health disparities work. Virtual agents provide an opportunity to explore and redefine trust. In this case, being perceived as friendly, with a kind voice, and overall appearance helped women engage the technology and messages being delivered.

### Expertise and authority

Preferences for authority, clothing and age played an important role in women’s assessments of expertise. Acceptable calibration of these cues required many tries. For example, researchers first thought patients would respond positively to a community health worker, and dressed the VHA in casual clothing, which was unacceptable. Scrubs were also less preferred compared to a VHA wearing a white medical coat. These findings provide an opportunity to assess how cues that confer expertise can be extended and communicated virtually. There is also an opportunity to assess how different cues confer authority on the VHA compared to cues that signal authority with real-life health providers.

### Navigability

When expectations about functionality, features, and movement through a technology are not met it can negatively affect acceptability judgments, and perceived usefulness. Subtitles, lack of a back button, ability to select multiple responses to questions, and presences of a clickable pause button all affected ease of use perceptions. Participants' discussions revealed that these cues worked together to shape perceptions. Expectancy violations, typo's in written sources, poor site design, poor visual appearance, and non-professional looking content can result in low acceptability. For our audience, details such as the size of subtitled text, synchronicity of movements with audio, were relevant features of this technology. Web-based interventions should be studied to enhance engagement and acceptability among an diverse older adults, to eliminate the potential of a digital divide among populations who can reap a number of benefits from novel health technologies.

### Limitations

While there was interest in customizing the app (e.g., real-time selection and individual tailoring of specific features), participants were not able to select their agent prior to the interaction. Other limitations include inability to assess perceptions of non-concordant VHAs. Additionally, participants who tested print prototypes were not included in exploratory statistical analysis. Finally, some cues (e.g., friendliness, clothing) were not explicitly asked on questionnaires and could not be triangulated with qualitative insights.

## Conclusions

This systematic assessment of adaptations to a VHA and features of web-based intervention promoting cancer screening defines acceptable cues based on Black women’s perceptions. Modifiable, non-verbal behaviors such as facial expressions, gaze, and gestures can improve acceptability, as can graphics, text subtitles, and audio content. For an ageing, minoritized population, previous experiences with medical care may affect perceptions of cues and overall acceptability of the technology. Remaining questions include what combination of cues trigger optimal engagement? The rapid expansion of health technologies provides opportunities to document key strategies for creating engaging, easy to use, and acceptable tools for rural, ageing, and minoritized populations.

## Data Availability

The datasets supporting the conclusions of this article are available upon reasonable request. Contact Janice L. Krieger, janicekrieger@ufl.edu for data requests.
